# Process optimization of jet electrodeposition Ni–Co–P alloy coating using response surface methodology and the tribological behavior of Ni–Co–P nanocomposite coatings

**DOI:** 10.1038/s41598-025-90163-4

**Published:** 2025-02-14

**Authors:** Yin Zhang, Yonggang Wang, Liang Yao, Lingling Chen

**Affiliations:** 1 School of Mechatronics Engineering, Anhui University of Science and Technology, Huainan, 232001 China; 2 The First Affiliated Hospital of Anhui, University of Science and Technology (Huainan First People’s Hospital), Huainan, 232007 China; 3 NARI Group Corporation (State Grid Electric Power Research Institute), NARI Technology Development Co. Ltd, Nanjing, 211106 Jiangsu China; 4 Office of Laboratory and Equipment Management, Anhui University of Science and Technology, Huainan, 232001 China

**Keywords:** Jet electrodeposition, Ni–Co–P composite coatings, Microhardness;, Wear resistance, Metals and alloys, Nanoparticles

## Abstract

A novel Ni–Co–P alloy coating and Ni–Co–P nanocomposite coating were prepared by jet electrodeposition. The influence of jet electrodeposition processing parameters on the microhardness and wear track width of the Ni–Co–P alloy coating was investigated. Additionally, the cross-section morphology, EDS spectra, XRD patterns, microhardness and wear resistance of the coatings under the optimum jet electrodeposition parameters were evaluated. The BBD analysis results revealed that the established mathematical model was reliable. Furthermore, the optimum Ni–Co–P alloy coating parameters optimized through the response surface method were as follows: jet voltage: 12.14 V, plating solution temperature: 61.60 °C, reciprocating sweep speed: 173.19 mm·s^−1^, jet gap: 2.05 mm, pulse frequency: 4.06 kHz and duty cycle: 0.81. Under the optimum jet electrodeposition parameters, the results revealed that the significant influence of nano BN(h) and Al_2_O_3_ particles on the coatings’ thickness, Co contents, crystallite size, microhardness and wear resistance of Ni–Co–P nanocomposite coating. In addition, compared with the Ni–Co–P alloy coating and Ni–Co–P–BN(h) nanocomposite coating, the Ni–Co–P–Al_2_O_3_ composite coating exhibited a larger thickness (18.16 µm) and Co element contents (39.51 wt·%), a smaller crystallite size (16.440 nm), a higher microhardness (676.5 HV_0.2_), a more excellent wear resistance (402.9 µm).

## Introduction

Corrosion and wear are well-established phenomena that occur spontaneously due to the combined effects of chemical, physical, and physicochemical factors^1^. These processes result in the gradual deterioration of the geometric structure of metal components, increased material wear, shortened service life of equipment, and heightened risk of catastrophic failures. As such, corrosion and wear represent significant challenges for industries, leading to annual economic losses amounting to billions of dollars worldwide^2^. To date, surface films have been widely studied for their ability to serve as protective barriers, isolating the substrate material from corrosive environments and improving the wear resistance of materials^3,4^. Consequently, numerous studies have been conducted to mitigate corrosion and wear damage. Various methods have been developed and refined, including magnetron sputtering^5^, chemical etching^6^, chemical vapor deposition^7^, thermal spraying^8^, thermal oxidation^9^, anodization^10^, electrophoresis^11^, electrodeposition^12^, and jet electrodeposition^13^. Each of these techniques offers unique advantages in enhancing surface properties, thereby extending the functional lifespan of metallic components and improving their performance in harsh operational environments.

Jet electrodeposition (JED) is an advance and highly efficient manufacturing technology capable of achieving higher overpotential and producing metallic nano-scaled microstructures with exceptional performance characteristics during the deposition process^14,15^. For instance, Tian et al.^15^ fabricated Ni coatings using JED and reportedan average grain size of 13.7 nm at a current density of 39.8 A‧dm^−2^. Zhang et al.^16^ observed optimal wear resistance in Co–Ni–Cr_3_C_2_ nanocomposite coatings at a current density of 40 A‧dm^−2^. Similarly, Ye et al.^17^ demonstrated that deposition rates increased with increasing jet voltage. Xia et al.^18^ investigated the effects of jet rate on Ni–TiN coating and found that the microhardness reached 876.2 HV at a jet rate of 3 m/s. Tian et al.^19^ studied the grain sizes of Co–Ni alloy coating under varying bath flow conditions and observed that increasing bath flow rates resulted in smaller grain sizes. Cui et al.^20^ concluded that increasing the diameter of the anode nozzle enhanced the flow rate of the plating solution. Qiao et al.^21^ investigated the effects of jet speed and plating solution temperature on Co^2+^ ion content in Ni–Co coatings. Furthermore, Jiang et al.^22–24^ demonstrated that the application of a magnetic field significantly improved both the corrosion resistance and mechanical properties of JED coatings. Wang et al.^25^ identified optimal jet electrodeposition parameters for preparing Co–Cr_3_C_2_ coatings and reported that the mass fraction of Cr_3_C_2_ was significantly affected by gun movement speed. Mridul et al.^26^ developed mathematical models capable of predicting the relationship between deposition rate and jet gap. Therefore, the optimal selection of JED parameters can accelerate deposition rates, refine grain sizes, enhance microhardness, and improve both wear and corrosion resistance. However, much of the existing research has focused on individual parameter effects, often neglecting the interactions among multiple combinations of electrodeposition parameters. Additionally, comprehensive mathematical models for simultaneous optimization of multiple responses are lacking, thereby limiting the ability to achieve multiple objectives for simultaneous optimization.

Ni–Co–P alloy coatings have demonstrated exceptional wear resistance, hardness, saturation magnetization, and polarization resistance. Safavi et al.^27^ reported that Ni–Co–P alloy coatings exhibit better corrosion resistance compared to Ni–Co coatings, regardless of the applied current density. Li et al.^28,29^ found that jet speed, jet voltage, and pulse parameters affected the microhardness and corrosion resistance of the coating by altering the Co content. Furthermore, Zhang et al.^30–32^ conducted single-factor experiments to analyze the effects of jet voltage, jet gap, temperature, pulse frequency, and duty cycle on the wear and seawater corrosion resistance of the Ni–Co–P alloy coatings. The response surface methodology (RSM) offers a robust approach to parameter optimization by combining experimental design and model building. The RSM establishes mathematical relationships between design variables and response values using multiple regression equations, thereby enabling the identification of optimal parameter settings based on specific objectives. Wang et al.^33^ investigated the effect of spraying parameters on the microstructure and the bonding strength of Y2O3 coatings based on the RSM, the results demonstrated that the RSM could be effectively adopted to design the atmospheric plasma spraying (APS) coating. Yazdani et al.^34^ utilized the RSM to optimize the conditions of electroless Ni–B nano-diamond baths, which was reliably supported by experimental results of a highest polarization resistance and the thickness. It can be concluded that the RSM is an accurate and welcomed experimental design method to design the preparation parameters. Thus, based on the results of previous studies, exploring the interaction effects of JED parameters using RSM could provide valuable insights into determining optimal process conditions, thereby enhancing the wear resistance and overall performance of Ni-Co-P alloy coatings.

Al_2_O_3_ nanoparticles are characterized by high hardness, wear and corrosion resistance. Chiu et al.^35^ reported that incorporating Al_2_O_3_ nanoparticles significantly enhanced the wear resistance of coatings compared to pure nickel coating. Wang et al. ^36^ investigated the effect of Al_2_O_3_ particle content on the structure and performance of electrobrush-plated Ni–Co coating. Similarly, Zhang et al.^37^ fabricated Ni–Co–W–Al_2_O_3_ coatings using direct current electrodeposition and reported that the incorporation of Al_2_O_3_ particles markedly improved wear resistance compared to pure Ni–Co–W coatings. Meanwhile, BN(h) (hexagonal boron nitride) nanoparticles are characterized by low friction coefficient, superior lubricity, and enhanced tribological properties^38^. Chen et al.^39^ observed that addition of h-BN significantly reduced the coefficient of friction and wear rate in the composite coatings. Kandadai et al.^40^ reported that the presence of h-BN layers improved the corrosion resistance. Tian et al.^41^ reported that an armor material made of h-BN had better impact resistance. Building on these findings, Ni–Co–P–BN(h) and Ni–Co–P–Al_2_O_3_ nanocomposite coatings were fabricated using jet electrodeposition under optimized process parameters. This study investigated the effect of BN(h) and Al_2_O_3_ nanoparticles on the microhardness and wear resistance of the coatings.

In this paper, the influence of jet electrodeposition processing parameters on the microhardness and wear track width of the Ni–Co–P alloy coating was investigated. Comprehensive evaluations were conducted, including cross-section morphology analysis, energy dispersive spectroscopy (EDS) spectra, X-ray diffraction (XRD) patterns, microhardness measurements, and wear resistance tests under optimal jet electrodeposition conditions. Furthermore, the effect of BN(h) and Al_2_O_3_ nanoparticles on key coatings characteristics, such as thickness, Co content, crystallite size, microhardness, and wear resistance of the coating were analysed under the optimum jet electrodeposition parameters. These investigations provided insights into the synergistic effects of the nanoparticles and the potential for optimizing nanocomposite coatings for enhanced mechanical and tribological performance.

## Experimental procedures

### Experimental setup

**Fig.1:**
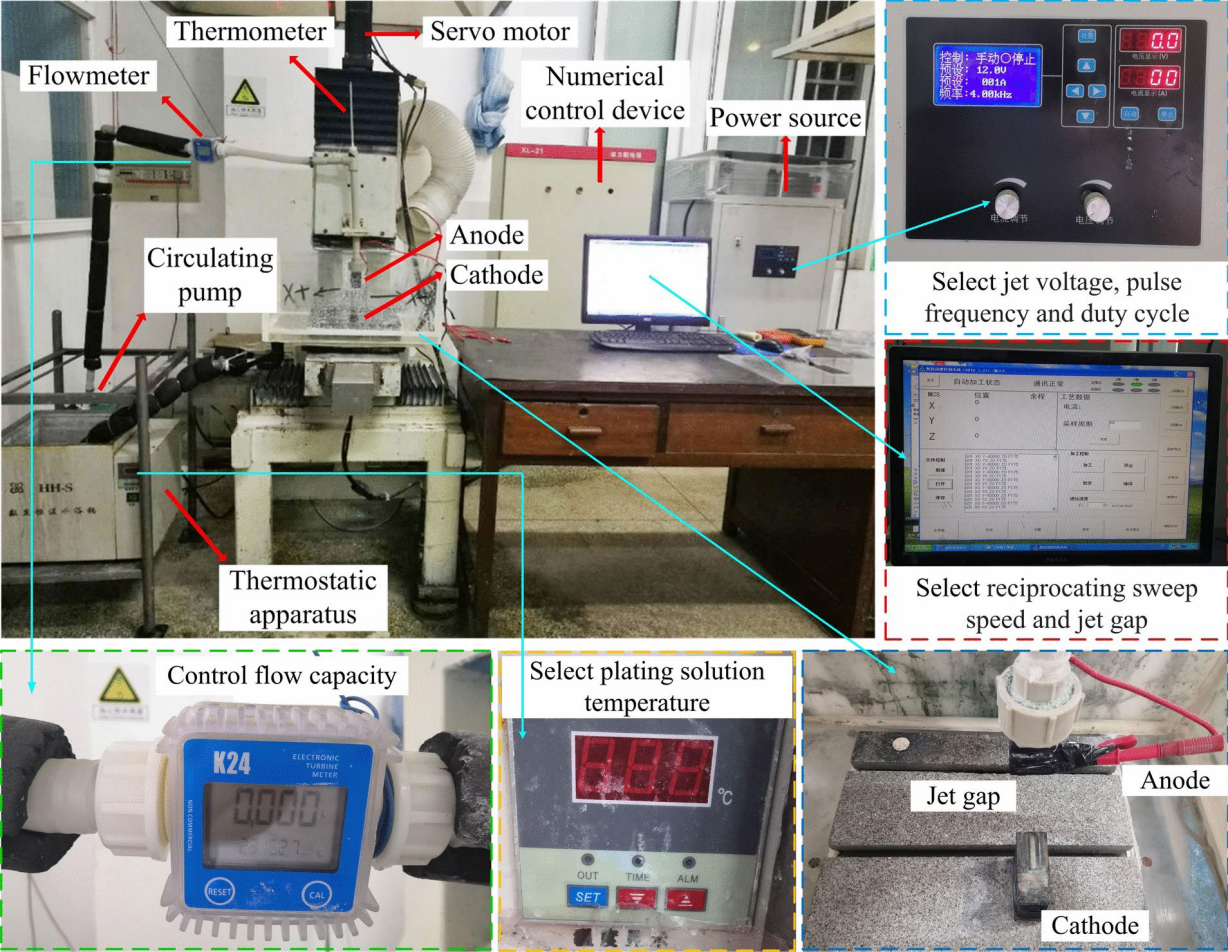
The numerically controlled jet electrodeposition experimental setup.

The experimental setup for numerically controlled jet electrodeposition (JED) is depicted in Fig. 1. The JED process began with the thermostatic apparatus, which heated the plating solution to a predefined temperature. The heated plating solution was then transferred by a circulating pump and impinged on the cathode surface at high velocity. A flowmeter and thermometer provided real-time measurements of the plating solution’s flow rate and temperature, respectively. After impingement, the plating solution recirculated back to the thermostatic apparatus. The anodic nozzle, designed with a rectangular shape, oscillated along the Y-axis in a continuous reciprocating motion. The reciprocating sweep speed between the anode (nozzle) and the cathode (substrate) was controlled by the servo device. Jet deposition occurred within the impingement area when an appropriate voltage was applied via power source. The setup was equipped with features to regulate critical process parameters. Concurrently, the power source allowed for the precise setting of jet voltage, pulse frequency, and duty cycle. Overall, the jet electrodeposition experimental setup comprised of a thermostatic apparatus, a power source, a circulating pump, a servo device, and machine accessories. The enhanced deposition rate achieved during JED was attributed to the continuous high-speed flow and efficient transport of metal ions within the plating solution, under the combined action of an electric field and flow field.

### Substrate materials and coating preparation

Steel C1045 materials (with a size of 30 × 8 × 7 mm) purchased from Suzhou Co. (Jiangsu, China) were used as the substrate of the experiment. Table 1 indicated the chemical composition of steel C1045 materials. Jet electrodeposition was carried out using a sulphate bath. The Ni–Co–P alloy coating were deposited from an aqueous solution containing 200.0 g·L^−1^ NiSO_4_·6H_2_O (supply Ni^2+^ ions), 20.0 g·L^−1^ CoSO_4_·7H_2_O (supply Co^2+^ ions), 30.0 g·L^−1^ NiCl_2_·6H_2_O (conducting salt), 30.0 g·L^−1^ H_3_BO_3_ (buffering agent), 20.0 g·L^−1^ H_3_PO_3_ (supply P source), 60.0 g·L^−1^ C_6_H_8_O_7_·H_2_O (complexant), 0.08 g·L^−1^ CH_3_(CH_2_)_11_SO_4_Na (surfactant), 0.02 g·L^−1^ CH_4_N_2_S (stabilizing agent). The average sizes of BN(h) and Al_2_O_3_ are 50 nm and 30 nm, respectively, which were purchased from Hefei ZhongHang Nanometer Technology Development Co., Ltd. Before adding into the Ni–Co–P bath, the 8.0 g·L^−1^ BN(h) and 8.0 g·L^−1^ Al_2_O_3_ powder was washed with deionized water. Then ultrasonic treatment was applied for 30 min. Finally, the dispersed BN(h) and Al_2_O_3_ suspensions were separately added to the Ni–Co–P plating solution, stirred for 2 h and dispersed by ultrasonic treatment for 30 min, and a composite solution was obtained.

**Table 1:** Composition of steel C1045 substrate materials.

C/(wt.%)	Si/(wt.%)	Cr/(wt.%)	Ni/(wt.%)	Mn/(wt.%)	P/(wt.%)	Cu/(wt.%)	S/(wt.%)
0.46	0.27	0.05	0.04	0.59	0.02	0.05	0.02

### Design of the experiment

Open Design Expert software and navigate to Box-Behnken design (BBD) under response surface methodology. Factors *X*_1_, *X*_2_, *X*_3_, *X*_4_, *X*_5_ and *X*_6_ represent jet voltage (V), plating solution temperature (°C), reciprocating sweep speed (mm·s^−1^), jet gap (mm), pulse frequency (kHz) and duty cycle, respectively. One response values Y_1_ represent microhardness (HV_0.2_). Furthermore, the aim is to maximize Y_1_ design. The level and range of factors are shown in Table 2. Each level consists of three sections: low, medium, and high section which correspond to –1, 0, and 1, respectively. In this model, 54 experiments are required to study the six variables. Six replicates were performed at the center points (level 0) of the six variables to estimate the error. Considering the equipment requirements and the quality of coating, the jet voltage (*X*_1_) were selected in the range of 10–14 V, plating solution temperature (*X*_2_) in the range of 55–65 °C, reciprocating sweep speed (*X*_3_) in the range of 150–200 mm·s^−1^, jet gap (*X*_4_) in the range of 1.5–2.5 mm, pulse frequency (*X*_5_) in the range of 3–5 kHz and duty cycle (*X*_6_) in the range of 0.7–0.9.

**Table 2:** Levels and range of factors in experimental design.

Variable factors	Encoding values	Scope and level		
		**–**1	0	1
Jet voltage *X*_1_ (V)	*x*_1_	10	12	14
Plating solution Temperature *X*_2_ (°C)	*x*_2_	55	60	65
Reciprocating sweep Speed *X*_3_ (mm·s^−1^)	*x*_3_	150	175	200
Jet gap *X*_4_ (mm)	*x*_4_	1.5	2.0	2.5
Pulse frequency *X*_5_ (kHz)	*x*_5_	3	4	5
Duty cycle *X*_6_ (–)	*x*_6_	0.7	0.8	0.9

### Design of the experiment

The cross-sectional morphology of the coatings was analyzed using scanning electron microscopy (SEM; Quanta FEG 250, FEI Instruments, USA). The chemical composition and distribution of chemical elements within the composite coatings were analyzed using energy-dispersive spectroscopy (EDS; X Flash Detector 5030, BRUKER, Karlsruhe, Germany). The crystallographic structure of the composite coating was observed using X’Pert Power X-ray diffraction (XRD; PANalytical B.V., Almelo, Holland) with CuKα radiation. The XRD parameters included a voltage of 40 kV, a step size of 0.02°, and an incidence angle of 2θ = 20°–90°. Additionally, the grain size of the composite coatings was calculated using the Debye–Scherrer equation.

Micro-hardness testing of the coatings was carried out using a Struers Duramin-40 (US) microhardness tester (Ballerup, Copenhagen, Denmark). A 200 g force was applied with a dwell time of 15 s during loading. Micro-hardness experiments were conducted at 5 distinct points to ensure accuracy of results. The average value was recorded as the final result. Dry sliding wear tests were performed using a high frequency reciprocating wear test machine (Lanzhou Zhongke Testing Instrument Co., Ltd.). The friction mode was reciprocating dry friction, with a GCr15 grinding ball of 3 mm diameter serving as the counterface material. Test conditions were standardized with a single friction stroke of 4 mm, a friction time of 30 min, a friction frequency of 2 Hz, and a normal loading force of 3.2 N. After each wear test, the coatings were ultrasonically cleaned in an ultrasonic cleaner to remove debris and contaminants. Laser scanning confocal microscopy (LSCM, OLS4000, OLYMPUS, Tokyo, Japan) was employed to characterize the worn grooves of the coatings.

## Results and discussion

### Experimental test results

From the Table 3, it can be observed that the best Ni–Co–P coating was deposited with 12 V jet voltage, 60 °C plating solution temperature, 175 mm·s^−1^ reciprocating sweep speed, 2.0 mm jet gap, 4 kHz pulse frequency and 0.8 duty cycle. It exhibited a microhardness of 656.7 HV_0.2_.

**Table 3:** Experimental results.

Run	Mode	*X*_1_ / (V)	*X*_2_ / (°C)	*X*_3_ / (mm·s^−1^)	*X*_4_ / (mm)	*X*_5_ / (kHz)	*X*_6_ / ( −)	*Y*_1_ / (HV_0.2_)
1	0 + + 0 − 0	12	65	200	2.0	3	0.8	642.5
2	000000	12	60	175	2.0	4	0.8	652.8
3	0 + 00 − −	12	65	175	2.0	3	0.7	630.9
4	+ 0 − 00 +	14	60	150	2.0	4	0.9	610.3
5	− 0 + 00 +	10	60	200	2.0	4	0.9	602.6
6	− 00 − − 0	10	60	175	1.5	3	0.8	604.5
7	0 + 00 + −	12	65	175	2.0	5	0.7	622.3
8	− 0 − 00 +	10	60	150	2.0	4	0.9	608.6
9	000000	12	60	175	2.0	4	0.8	656.6
10	0 + − 0 + 0	12	65	150	2.0	5	0.8	648.2
11	0 − − 0 + 0	12	55	150	2.0	5	0.8	632.7
12	− 00 + − 0	10	60	175	2.5	3	0.8	613.8
13	00 + + 0 −	12	60	200	2.5	4	0.7	625.2
14	− 00 − + 0	10	60	175	1.5	5	0.8	603.3
15	0 − + 0 + 0	12	55	200	2.0	5	0.8	597.8
16	+ 00 − − 0	14	60	175	1.5	3	0.8	616.2
17	000000	12	60	175	2.0	4	0.8	656.5
18	0 − 00 − −	12	55	175	2.0	3	0.7	620.7
19	0 − − 0 − 0	12	55	150	2.0	3	0.8	594.9
20	+ + 0 + 00	14	65	175	2.5	4	0.8	618.9
21	0 − 00 + −	12	55	175	2.0	5	0.7	620.4
22	+ − 0 + 00	14	55	175	2.5	4	0.8	610.2
23	0 − 00 + +	12	55	175	2.0	5	0.9	619.3
24	00 + − 0 +	12	60	200	1.5	4	0.9	624.6
25	000000	12	60	175	2.0	4	0.8	656.7
26	000000	12	60	175	2.0	4	0.8	655.2
27	− − 0 − 00	10	55	175	1.5	4	0.8	589.7
28	00 − − 0 +	12	60	150	1.5	4	0.9	629.8
29	0 − + 0 − 0	12	55	200	2.0	3	0.8	623.2
30	+ − 0 − 00	14	55	175	1.5	4	0.8	605.8
31	− + 0 − 00	10	65	175	1.5	4	0.8	612.5
32	0 − 00 − +	12	55	175	2.0	3	0.9	616.2
33	00 + − 0 −	12	60	200	1.5	4	0.7	627.6
34	− 00 + + 0	10	60	175	2.5	5	0.8	615.4
35	+ 00 + + 0	14	60	175	2.5	5	0.8	620.9
36	0 + 00 + +	12	65	175	2.0	5	0.9	638.8
37	+ 00 − + 0	14	60	175	1.5	5	0.8	622.7
38	− + 0 + 00	10	65	175	2.5	4	0.8	617.4
39	− 0 + 00 −	10	60	200	2.0	4	0.7	596.3
40	0 + − 0 − 0	12	65	150	2.0	3	0.8	616.5
41	00 − + 0 +	12	60	150	2.5	4	0.9	632.2
42	+ 0 + 00 −	14	60	200	2.0	4	0.7	613.7
43	00 − + 0 −	12	60	150	2.5	4	0.7	628.5
44	+ + 0 − 00	14	65	175	1.5	4	0.8	624.7
45	− 0 − 00 −	10	60	150	2.0	4	0.7	595.8
46	+ 0 − 00 −	14	60	150	2.0	4	0.7	613.4
47	+ 00 + − 0	14	60	175	2.5	3	0.8	616.7
48	00 + + 0 +	12	60	200	2.5	4	0.9	628.5
49	0 + 00 − +	12	65	175	2.0	3	0.9	634.2
50	+ 0 + 00 +	14	60	200	2.0	4	0.9	615.3
51	0 + + 0 + 0	12	65	200	2.0	5	0.8	614.5
52	00 − − 0 −	12	60	150	1.5	4	0.7	628.6
53	− − 0 + 00	10	55	175	2.5	4	0.8	600.2
54	000000	12	60	175	2.0	4	0.8	652.9

### Analysis of technological parameters on the microhardness

To determine a critical point from the table data, quadratic terms are included in the polynomial function. BBD is a three-level factorial design which can be used with the quadratic Eq. (1)^42,43^:

1 $$Y = b_{0} + \sum\limits_{i = 1}^{k} {b_{i} } x_{i} + \sum\limits_{i = 1,j = 1}^{k} {b_{ij} } x_{i} x_{j} + \varepsilon$$

where Y was the response value, b_0_ was the constant coefficient, *k* represents the number of factors,${\beta }_{i}$ b_i_ was the linear parameter coefficients,${x}_{i}$ *x*_i_ was the variables,${\beta }_{ij}$ b_ii_ represents the coefficients of the quadratic perimeter, b_ij_ was the interaction parameter coefficients, *x*_j_ was processing parameters, ε was the residual associated to the experiments ${\beta }_{ii}$.

The microhardness value obtained from the response value using BDD design is given by Eq. (2):

2 $$\begin{gathered} Y_{1} \, = \,{-}4088.49\, + \,180.76X_{1} \, + \,61.89X_{2} \, + \,9.40X_{3} \, \hfill \\ + \,154.39X_{4} \, + \,163.19X_{5} \, + \,1069.69X_{6} {-}0.16X_{1} X_{2} \, \hfill \\ + \,0.027X_{1} X_{3} {-}2.47X_{1} X_{4} \, + \,0.64X_{1} X_{5} {-}12.88X_{1} X_{6} {-}0.79X_{2} X_{4} \hfill \\ {-}0.19X_{2} X_{5} \, + \,6.35X_{2} X_{6} {-}0.61X_{3} X_{5} \, + \,22.0X_{4} X_{6} \, + \,20.75X_{5} X_{6} {-}6.70X_{1}^{2} \hfill \\ {-}0.51X_{2}^{2} {-}0.02X_{3}^{2} {-}22.73X_{4}^{2} {-}8.44X_{5}^{2} {-}862.36X_{6}^{2} \hfill \\ \end{gathered}$$

The process parameters (X_1_, X_2_, X_3_, X_4_, X_5_, X_6_) corresponding to a single response were evaluated and a regression equation developed. This obtained the optimal combination for each response. To determine the overall performance of the entire experiment design, the six regression equations from the six separate responses were combined. Microhardness target response Y_1_ is a maximum value. The target values Y_1_ cannot be 0, and the x-axis values for the factors X_1_, X_2_, X_3_, X_4_, X_5_ and X_6_ cannot be negative, so constraints were set as shown in Eq. (3):

Y_i_ ≥ 0.

3 $$1\, \le \,x_{i} \, \le \,1(i\, = \,1, \, 2, \, 3, \, 4, \, 5, \, 6;xshows \, the \, x - axis \, values \, for \, the \, factorsX_{1} , \, X_{2} , \, X_{3} , \, X_{4} , \, X_{5} and \, X_{6} )$$

The predicted optimized processing parameters for jet electrodeposition were a jet voltage of 12.14 V, plating solution temperature of 61.60 °C, reciprocating sweep speed of 173.19 mm·s^−1^, jet gap of 2.05 mm, pulse frequency of 4.06 kHz, and a duty cycle of 0.81. Under these optimized conditions, the predicted responses included microhardness of 656.7 HV_0.2_.

Data for the microhardness is shown in Table 4. The analysis indicates that the F-value is 117.16 and the P-valuewas less than 0.0001, confirming a significant relationship in the regression equation of the model. The proposed F-ratio was 1.78. The F value gives a check to the significance, whereas P value is used to find out whether the probability of F value surpass the calculated value due to noise^44^. The p-value parameter measures the significance of the proposed model where a p-value less than 0.05 confirms the significance of the model^45^. The lower the value of P, the more significant the regression coefficient. As such, 0.01 < P < 0.05 indicates the factors influence on the results is extremely considerable, and P > 0.05 shows that the factors influence is more significant on the test results. A P-value higher than 0.005 shows that the lack of fit is insignificant relative to the pure error. The closer the determination (R^2^) coefficient value is to 1, the better the model fit is^46^. The determination (R^2^) coefficient was 0.9889, signifying that the regression model explains 98.89% of the variability in the experimental data. In addition, the adjusted decision coefficient R_adj_^2^ ${\text{R}}_{\text{adj}}^{2}$ was 98.05% and the predicted decision coefficient was 97.28%. The difference between the adjusted decision coefficient and the predicted decision coefficient was less than the required value of 0.2, indicating a good coincidence^47^. These statistic metrics confirm that the quadratic equation provides a high degree of fit. Consequently, the microhardness response model demonstrated high credibility, and xan be reliably used to predict the microhardness of coatings with precision.

**Table 4:** Variance analysis after optimization of microhardness response model.

Parameter	Sum of squares	Degrees of freedom	Mean square	F value	P value	Significance
Model	15,059.78	23	654.77	117.16	< 0.0001	highly significant
X_1_	690.15	1	690.15	123.50	< 0.0001	highly significant
X_2_	1508.92	1	1508.92	270.01	< 0.0001	highly significant
X_3_	31.97	1	31.97	5.72	0.0232	significant
X_4_	59.85	1	59.85	10.71	0.0027	significant
X_5_	28.16	1	28.16	5.04	0.0323	significant
X_6_	57.04	1	57.04	10.20	0.0033	significant
X_1_ X_2_	19.22	1	19.22	3.44	0.0735	–
X_1_ X_3_	14.58	1	14.58	2.61	0.1165	–
X_1_ X_4_	97.51	1	97.51	17.45	0.0002	significant
X_2_ X_4_	31.20	1	31.20	5.58	0.0248	significant
X_1_ X_5_	13.26	1	13.26	2.37	0.1339	–
X_2_ X_5_	15.01	1	15.01	2.68	0.1116	–
X_3_ X_5_	1888.05	1	1888.05	337.85	< 0.0001	highly significant
X_1_ X_6_	53.04	1	53.04	9.49	0.0044	significant
X_2_ X_6_	80.64	1	80.64	14.43	0.0007	significant
X_4_ X_6_	9.68	1	9.68	1.73	0.1981	–
X_5_ X_6_	34.44	1	34.44	6.16	0.0189	significant
X_1_^2^	7391.44	1	7391.44	1322.66	< 0.0001	highly significant
X_2_^2^	1659.70	1	1659.70	296.99	< 0.0001	highly significant
X_3_^2^	1655.35	1	1655.35	296.21	< 0.0001	highly significant
X_4_^2^	332.06	1	332.06	59.42	< 0.0001	highly significant
X_5_^2^	732.73	1	732.73	131.12	< 0.0001	highly significant
X_6_^2^	764.91	1	764.91	136.87	< 0.0001	highly significant
Lack of fit	150.74	25	6.03	1.78		
Pure error	16.90	5	3.38	–		
Total error	167.64	30	5.59	0.27		
Correction total	15,227.42	53				
R^2^ = 0.9889, R_adj_^2^ = 0.9805, R_pred_ = 0.9728						

**Fig.2:**
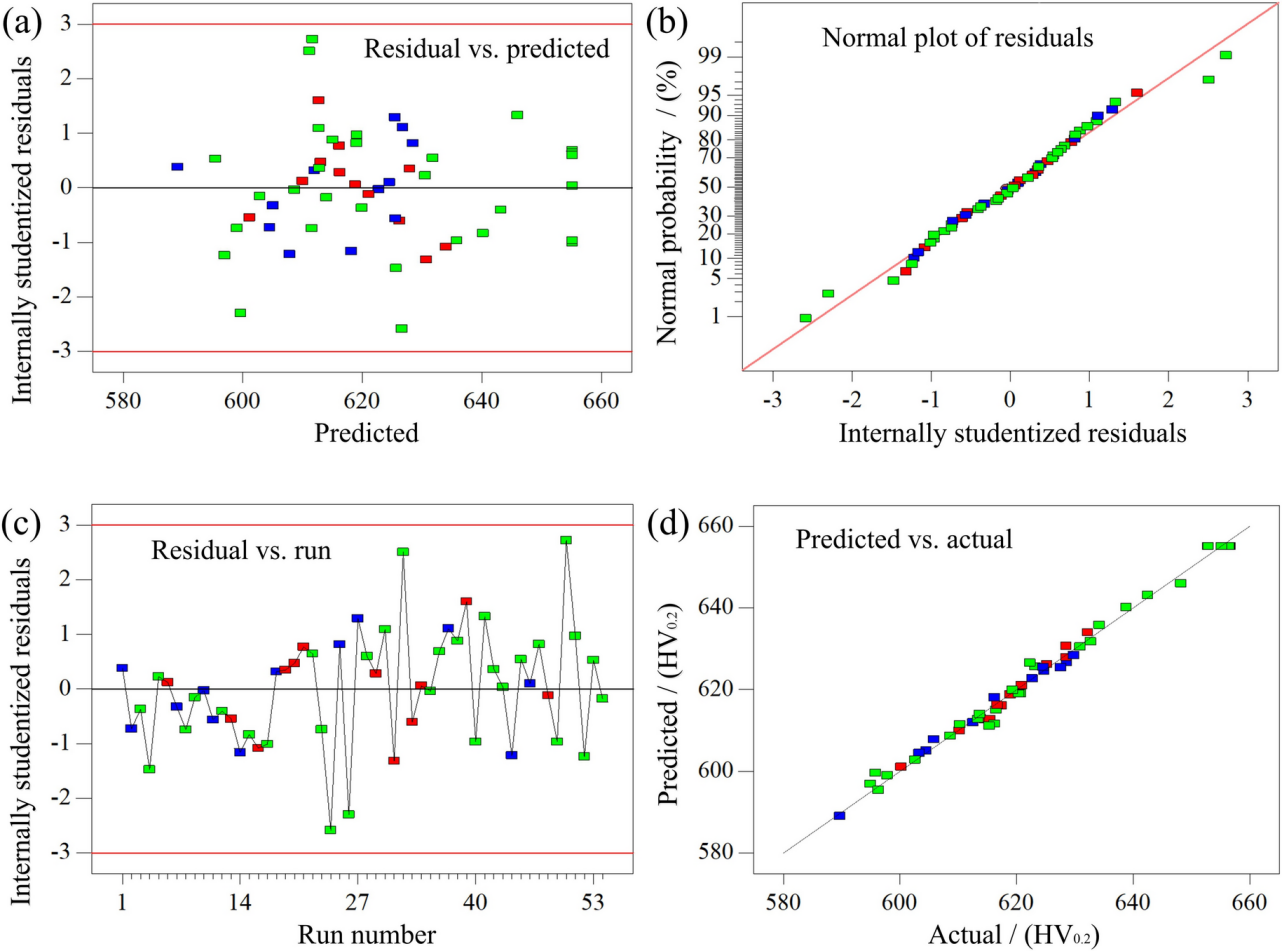
Residual diagram of microhardness: (**a**) residual vs. predicted; (**b**) normal of residuals; (**c**) residual vs. run; (**d**) analysis diagram of predicted and measured values of Y_1_.

Further testing of the microhardness response model was conducted, and the residual plot of the optimized microhardness response model is shown in Fig. 2. Figure 2 (a) shows the relationship between the residuals and predicted microhardness values. Notably, the residuals exhibited good fitting and reasonable relevance between the predicted and residual values, as all data points fell within the acceptable range of − 3.0 to + 3.0. This indicates a reasonable fit and strong relevance between the predicted values and residuals, consistent with findings reported in existing literature^48^. The scatter distribution of the residuals of the distribution in the microhardness response model appeared irregular, yet fell entirely within the defined error bounds, further verifying the reliability and accuracy of the microhardness response model. Generally, it is essential to validate any developed model before utilizing it for response analysis or optimization^43^. The normal probability plot of studentized residuals is one of the most important diagnostics, as shown in Fig. 2 (b). The residuals align approximately along a straight line, satisfying the assumption of normality. This confirms that the optimized microhardness response model effectively captures the trends in the experimental data ^49,50^.

Figure 2 (c) shows the experimental sequence and residual plot of the microhardness response model. The residual values of the microhardness response model were uniformly distributed both above and below the zero-point coordinate axis, with no apparent clustering of positive or negative residuals after optimization^51^. Therefore, this uniform distribution model accurately represents the true microhardness values of nickel–cobalt-phosphorus alloy coatings. To further verify the reliability of the regression analysis model, a comparison of the calculated and actual results is plotted in Fig. 2 (d). Usually, the closer the scatter distribution lies on either side of the predicted line, the closer the predicted values were to the experimental values, indicating a higher level of model reliability. In this case, all the measured microhardness values were tightly clustered around the predicted line, with no anomalous data points. . This confirmed that the microhardness prediction model effectively explain and illustrate the relationship among the six influencing factors with high accuracy and reliability. This phenomenon was consistent with the conclusion reached by Yan et al.^52^. It is reported in literature that the predicted results are in good agreement with the measured data, and the error was maintained within 5%.

**Fig.3:**
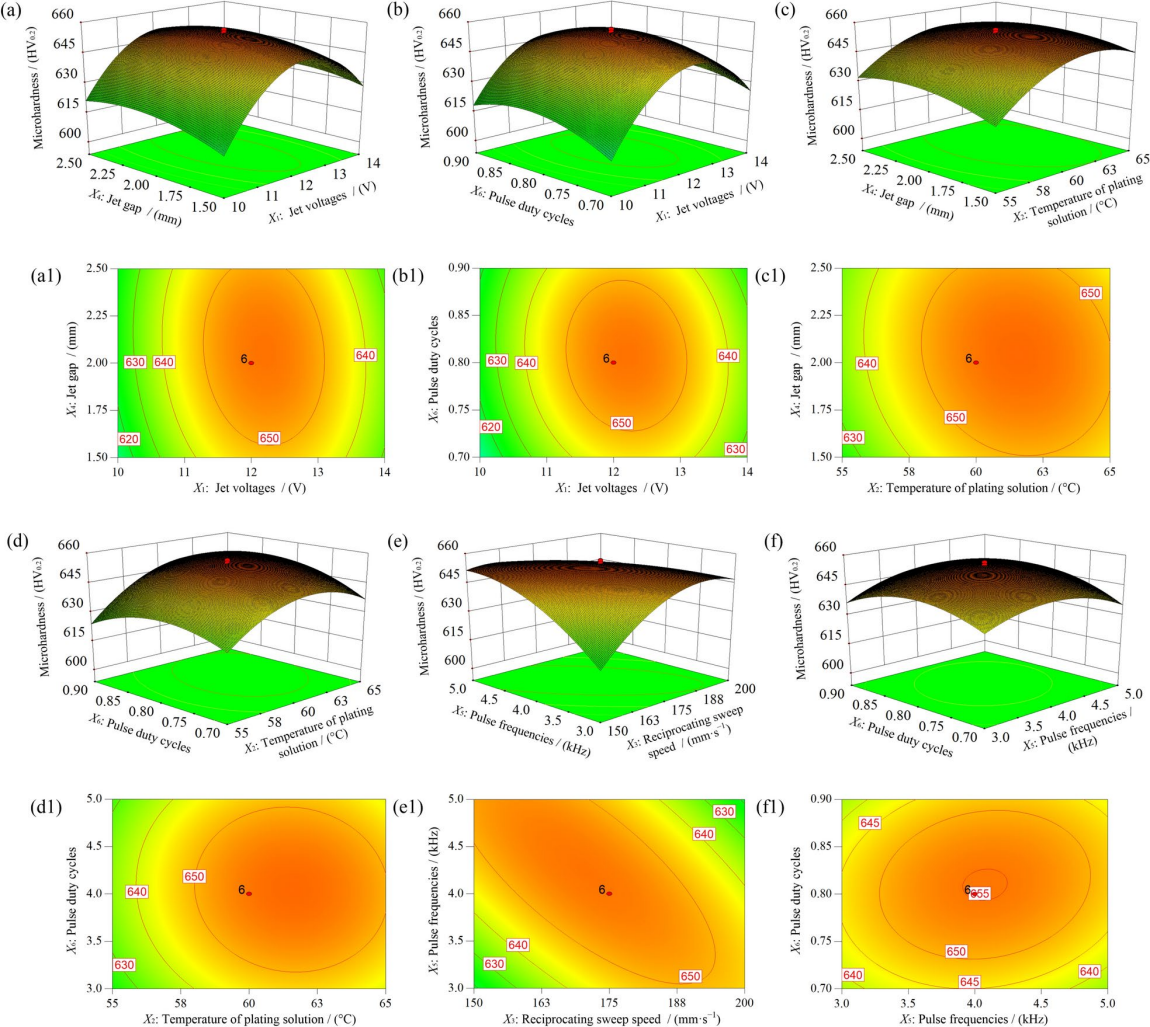
Response surface and contour plot of microhardness:

Three-dimensional (3D) plots were utilized to identify the optimal response regions and control the process parameters influencing the response. Each 3D plot depicted the relationship between each of the three responses against the significant process parameters while other process parameters were held constant. These visual 3D representations effectively presented the optimal factor regions of the experiment. The response surface in the vicinity of a critical point was characterized when the critical point on the graph had been identified. Figure 3 illustrates the response surface and contour plots for the microhardness model. It can be seen that all six independent variables influence the microhardness of the coatings. The observed nonlinear relationship was indicated by the significant curvature of the response surface, thereby forming a typical quadratic surface. The factors of jet voltage (X_1_), plating solution temperature (X_2_), reciprocating sweep speed (X_3_), jet gap (X_4_), pulse frequency (X_5_), and duty cycle (X_6_) all had poles, indicating that further optimization and analysis of microhardness was feasible. The degree of surface curvature of the response surface directly reflected the strength of interactions between independent variables, with higher curvature indicating stronger interactions. Additionally, a steep slope in a particular direction highlights a significant impact by the independent variable on the response results.

Figure 3 (a, a1) show the response surface and contour map for the interaction between jet voltage and jet gap, and their effects on the microhardness of the deposited Ni–Co–P coatings. Figure 3 (a) shows the response surface for the level of microhardness subject to varying the jet voltage and jet gap. The image reveals a maximum critical point, thereby indicating that the model maximizes the microhardness (*Y*_1_). It is evident that increasing jet voltage from level –1 (10 V) initially caused a sharp increase in the microhardness, which then transitioned to a more gradual increase until it reached an inflection point marking the maximum hardness near level 0 (12 V). Beyond this point, further voltage increases to level 1 (14 V) resulted in a decline in the microhardness. Similarly, as the jet gap increased from level -1 (1.5 mm) to 0 (2.0 mm), the microhardness of the coatings increased sharply, reaching an inflection point near level 0 (2.0 mm). Any further increase in the jet gap caused a decline in the microhardness. The contour map in Fig. 3 (a1) shows an elliptical region surrounding the critical point, indicating that the interaction between the jet voltage and jet gap was highly significant to the microhardness of the coatings.

To a certain extent, an increase in jet voltage enhanced the average current density on the cathode surface, thereby strengthening cathodic polarization effect and creating new nucleation sites. This accelerated the grain nucleation rate and improved microhardness of the alloy coating. However, excessive jet injection voltage strengthened the electric field, thereby accelerating consumption of nickel and cobalt ions in the solution. This resulted in insufficient concentration of metal ions near the cathode, reducing the grain refinement effect and ultimately decreasing the microhardness of the coating. It can also be seen that the response surface was steeper in the jet voltage direction, indicating that the impact of jet voltage on microhardness was greater compared to the jet gap. This observation aligns with the variance analysis results of the microhardness response model.

(a) response surface and (a1) contour plot of factor *X*_1_*X*_4_; (b) response surface and (b1) contour plot of factor *X*_1_*X*_6_; (c) response surface and (c1) contour plot of factor *X*_2_*X*_4_; (d) response surface and (d1) contour plot of factor *X*_2_*X*_6_; (e) response surface and (e1) contour plot of factor *X*_3_*X*_5_; (f) response surface and (f1) contour plot of factor *X*_5_*X*_6_.

Figure 3 (b) shows the response surface for the level of microhardness subject to varying jet voltage and duty cycle. When the duty cycle increases from level –1 (0.7) towards the critical point, there is a sharp rise in microhardness at first followed by a more gradual rise. Once the inflection point marking the critical point is reached, further increase in duty cycle causes a decrease in microhardness, gradual at first then sharply. The phenomenon can be attributed to increased duty cycle made the conduction time of power supply current also increased, which enhanced the overall electric field strength during the machining process and thus increases its microhardness. Further increased duty cycle, the current conduction of pulse power supply became too long, resulting in insufficient concentration of metal ions near the cathode, weakening the grain refinement effect, and instead reducing the microhardness. Figure 3 (b1) shows the contour map of the interaction between the two parameters. The contours form a distinct ellipse shape, indicating that the interaction between jet voltage and duty cycle on microhardness is significant.

Figure 3 (c) shows the interaction relationship between plating solution temperature and jet gap, and its effect on the microhardness of the deposited coating. As the plating solution temperature increased from level –1 (55 °C) to level 0 (60 °C), there was an increase in microhardness in a relatively uniform manner. At this point, the critical temperature was reached, and further increase in plating solution temperature beyond this level lead to a decrease in microhardness, as evidenced by a shift towards level 1 (65 °C). The increase in microhardness at lower plating solution temperatures can be attributed to enhanced metallic ion activity, which facilitated faster ion transfer to the cathode, thereby promoting effective deposition, grain nucleation, and grain refinement. This resulted in improved microhardness in the deposited coatings. However, at higher plating solution temperatures, the decrease in microhardness was attributed to a decrease in grain nucleation and an increase in porosity of the deposited coatings. The decrease can be associated with increase in evaporation rates which causes subsequent increase in energy consumption^53^. Figure 3 (c1) shows the contour map of the interaction between the two parameters. The contours form nearly circular profiles, suggesting that the interaction between plating solution temperature and jet gap on microhardness was relatively weak.

Figure 3 (d) shows the interaction between plating solution temperature and duty cycle, and its influence on microhardness. The microhardness increased with increase in plating solution temperature until a critical maximum point was reached, beyond which further increase in plating solution temperature lead to a decrease in microhardness. This phenomenon can be attributed to the enhanced diffusion rate of Ni and Co ions in the electrolyte as the plating solution temperature increased from 55 °C to 60 °C. This in turn facilitated metal ions transport to internal growth points, which refined the grain structure and improved the microhardness of the coating surface. However, an increase in gas bubble formation in the plating solution may have resulted in increased porosity of the coating, thereby reducing the microhardness. Figure 3 (d1) shows the corresponding contour map of the interaction between the two parameters. The contours form a distinct elliptical shape, indicating that the interaction between plating solution temperature and duty cycle on microhardness was strong.

Figure 3 (e, e1) show the response surface and contour map for the interaction between reciprocating sweep speed and pulse frequency on the hardness of the deposited coating. Within the experimental range, when the pulse frequency was held constant, the microhardness increased significantly with the reciprocating sweep speed then gradually stabilized. This increase can be attributed to a faster diffusion rate of metallic ions through the electrolyte bulk to the substrate surface as the reciprocating sweep speed increased from level –1 (150 mm·s^−1^) to the critical point. However, reducing the reciprocating sweep speed and pulse frequency resulted in a significant decrease in microhardness. The interaction curve between reciprocating sweep speed and pulse frequency on the microhardness model of the coating formed an elliptical shape, indicating a strong interaction between these two parameters. Finally, Fig. 3 (f, f1) show the response surface and contour map illustrating the effect of duty cycle and pulse frequency interactions on the hardness of the deposited Ni–Co–P coatings. It is evident that the surface curvature formed by the interaction between pulse frequency and duty cycle on the microhardness of the coating was relatively small, indicating that the interaction between the two was less significant compared to the other parameters.

### Structures and property of the coating deposited at the proposed optimization parameter

#### Cross-section morphology and EDS spectra of the coating

**Fig.4:**
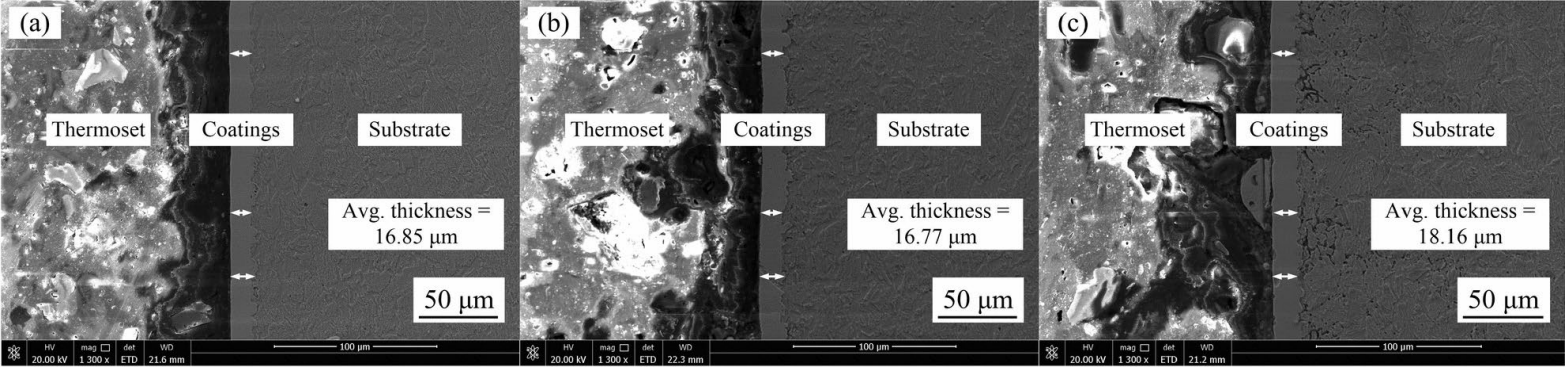
Cross-section morphologies of the coating prepared with the optimized processing parameters: (**a**) Ni–Co–P; (**b**) Ni–Co–P–BN(h); (**c**) Ni–Co–P–Al_2_O_3_.

The wear properties of the coating were notably affected by factors such as surface quality and thickness. Therefore, investigating the effect of nanoparticles on the thickness of nanocomposite coatings is essential for understanding their tribological behaviour. The cross-section morphologies of the coatings prepared under optimized processing parameter are presented in Fig. 4. As shown in Fig. 4 (a), the Ni–Co–P alloy coating exhibited no cracks at the interface with the steel C1045 substrate, indicating good adhesion, uniform transition, and distinct, straight interface. Additionally, the measured thickness of the Ni–Co–P alloy coating was 16.85 µm. In contrast, as seen in Fig. 4 (b) and (c), the thickness of the nanocomposite coatings exhibited slight variations, with the Ni–Co–P–BN(h) composite coating measuring 16.77 µm and the Ni–Co–P–Al2O3 composite coating showing an increased thickness of 18.16 µm.

Generally, the thickness of the coating obtained during electroplating is directly related to three variable factors: current density, current efficiency and circular telegram time. The higher the current density and the bigger current efficiency are, the longer the electroplating time is, and the thicker the coating is^54^. Therefore, the BN(h) and Al_2_O_3_ nanoparticles were added separately into Ni–Co–P basic plating solution, which may result in different cathode current efficiency at a constant current density and circular telegram time. This is the root reason that caused different the thickness. On the other hand, the BN(h) and Al_2_O_3_ nanoparticles themselves have different properties, it is commonly accepted that BN(h) belongs to the hexagonal system, which is supported by the van der Waals forces between the layers^55^, But Al_2_O_3_ particles are hard particles, which can resist plastic deformation and hinder abrasive movement.

**Fig.5:**
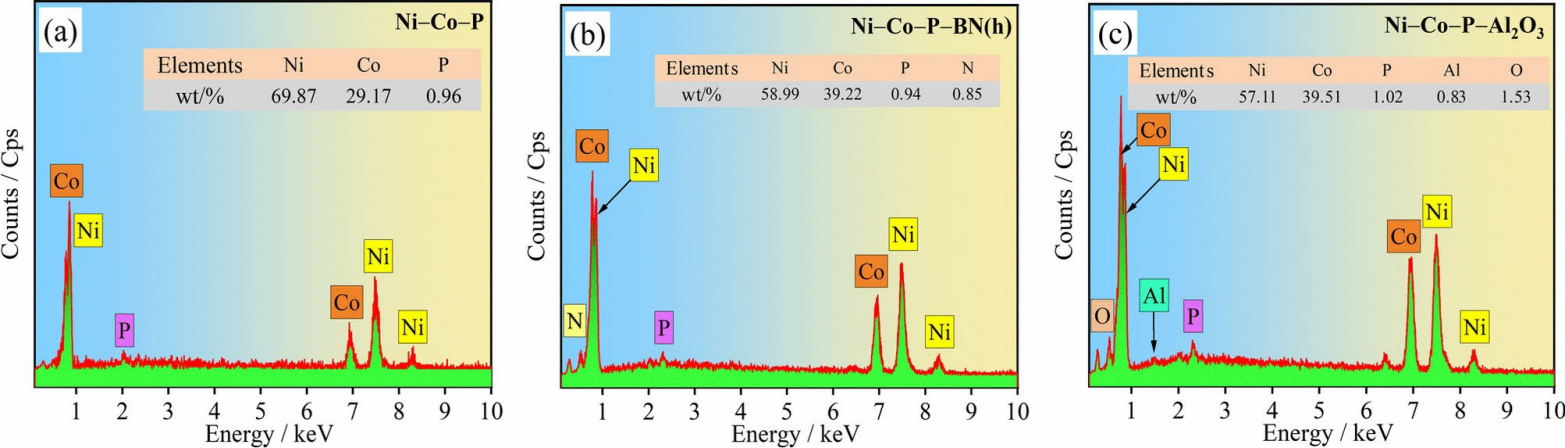
EDS spectra of the coatings prepared with the optimized processing parameters: (**a**) Ni–Co–P; (**b**) Ni–Co–P–BN(h); (**c**) Ni–Co–P–Al_2_O_3_.

The EDS spectra of the coatings prepared under optimized processing parameters are illustrated in Fig. 5. As shown in Fig. 5 (a), the mass fraction of Ni element, Co element, and P element in the coatings were 69.87 wt·%, 29.17 wt·% and 0.96 wt·%, respectively. The Co contents in the Ni–Co–P–BN(h) and Ni–Co–P–Al_2_O_3_ nanocomposite coating reached 39.22 wt·% and 39.51 wt·%, respectively. Furthermore, the mass fraction of BN(h) and Al_2_O_3_ nanoparticles in the coatings prepared via jet electrodeposition were 0.85 wt·%, and 0.83 wt·%, respectively. These results indicated a relatively low incorporation of nanoparticles into the coatings. Despite the low nanoparticle content, the addition of BN(h) and Al_2_O_3_ nanoparticles significantly affected the electrodeposition process. The nanoparticles affected current efficiency during electrodeposition, leading to changes in the Co element content within the Ni–Co–P–BN(h) and Ni–Co–P–Al_2_O_3_ nanocomposite coatings. These variations in composition subsequently impacted key material properties, including coating thickness, crystallite size, microhardness, and wear resistance.

#### Elemental mapping images of the coating

**Fig.6:**
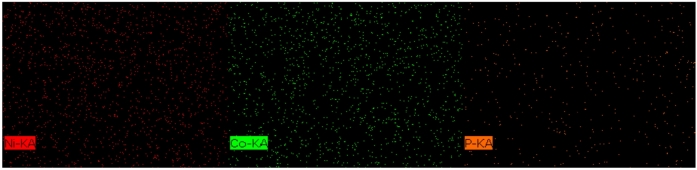
Elemental mapping images of Ni–Co–P alloy coating.

**Fig.7:**
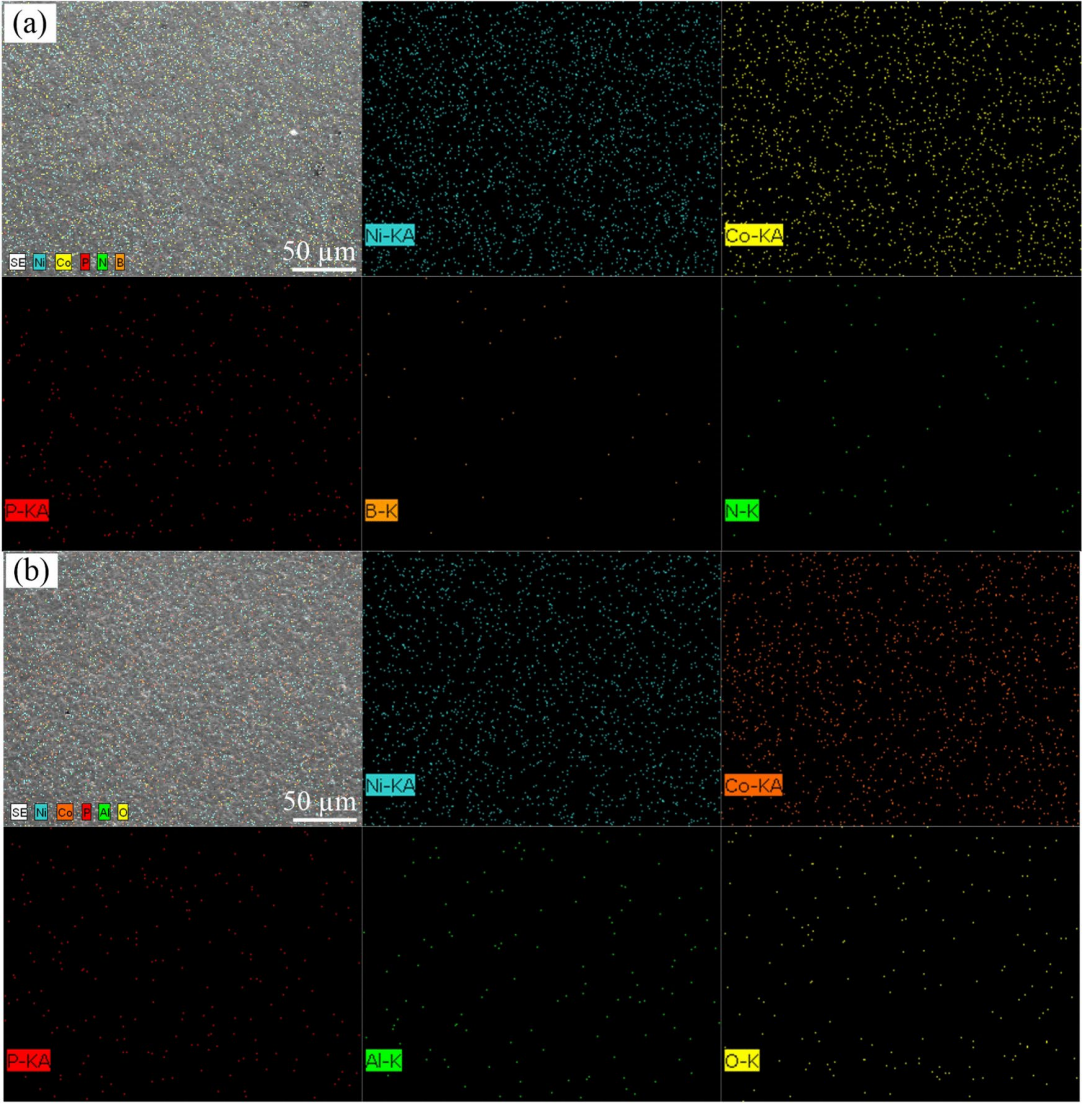
Elemental mapping images of Ni–Co–P nanocomposite coating: (a) Ni–Co–P–BN(h); (b) Ni–Co–P–Al_2_O_3_.

Figure 6 displays the elemental mapping images of the Ni–Co–P alloy coating prepared under optimized processing parameters. The images reveal that the surface of the Ni–Co–P alloy coatings’ produced via jet electrodeposition exhibited a uniform and dense structure without any cracks. Additionally, the elemental mapping confirmed that Ni, Co, and P were uniformly distributed throughout the deposited samples, indicating a consistent alloy composition throughout the sample. The elemental mapping images of the nanocomposite coatings surfaces are presented in Fig. 7. As shown in Fig. 7 (a) and (b), it is evident that the there was uniform distribution of Ni, Co, P, B, N, Al, and O across the coatings, which provides clear evidence of successful incorporation of the BN(h) and Al_2_O_3_ nanoparticles into the Ni-Co-P matrix.

#### XRD patterns of the coating

**Fig.8:**
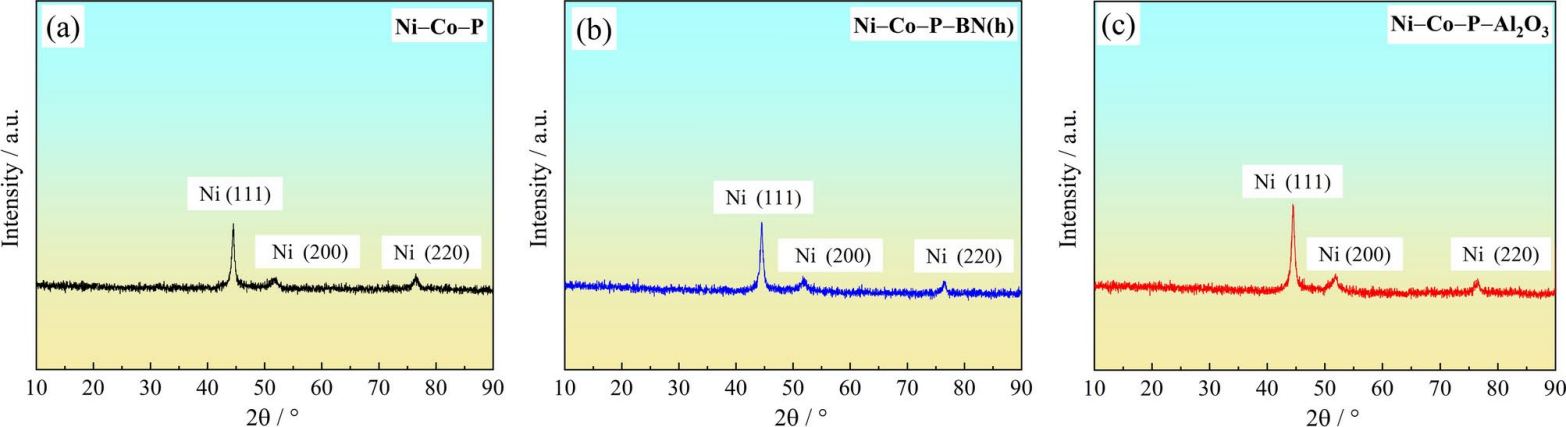
XRD patterns of the coatings prepared with the optimized processing parameters: (**a**) Ni–Co–P; (**b**) Ni–Co–P–BN(h); (**c**) Ni–Co–P–Al_2_O_3_.

Figure 8 shows the XRD patterns of the electrodeposited Ni–Co–P, Ni–Co–P–BN(h), and Ni–Co–P–Al_2_O_3_ composite coatings. As shown in Fig. 8 (a), according to PDF#04–0850, three distinct peaks were observed for the Ni–Co–P alloy coating patterns at 2θ = 44.383°, 51.932°, and 76.567°, which corresponded to the (111), (200), and (220) crystallographic face centred cubic (FCC) planes of Ni, respectively. This indicated that the deposited coatings exhibit a crystalline FCC structure which is characteristic of Ni–Co–P alloy. From Fig. 8 (b) and (c), it is evident that the incorporation of BN(h) and Al_2_O_3_ nanoparticles into Ni–Co–P alloy coating did not significantly reduce the intensity of the peaks corresponding to the Nickel (111) and (200) planes. Furthermore, no distinct shift in the peak positions was observed, which suggests that the addition of BN(h) and Al_2_O_3_ nanoparticles did not disrupt the crystalline structure of the Ni–Co solid solution. The XRD patterns displayed no distinct peaks for N and Al element, and this was attributed to the nanoscale size of the BN(h) and Al_2_O_3_ particles used in the experiment (50 nm and 30 nm, respectively), coupled with the relatively low concentrations of BN(h) particles and Al_2_O_3_ nanoparticles in the plating bath.

In this analysis, the crystallite size of the deposited coatings was determined using the integral peak width. The average particle particle size was calculated using the Debye–Scherrer equation. As shown in Table 5, the average crystallite size distribution of the Ni–Co–P alloy coating was 19.925 nm, indicating a nanocrystalline structure. The average crystallite size of the Ni–Co–P–BN(h) and Ni–Co–P–Al_2_O_3_ composite coating reached 16.687 nm and 16.440 nm, respectively. This indicated that the addition of BN(h) and Al_2_O_3_ nanoparticles in the coating resulted in a subsequent decrease in the crystallite size, with the nanocomposite coatings exhibiting smaller crystallite sizes compared to the pure Ni–Co–P alloy coating. This phenomenon was primarily affected by the Co content and the presence of nanoparticles in the coating. As illustrated in Fig. 5, the incorporation of BN(h) and Al_2_O_3_ nanoparticles in the coatings significantly influenced the Co content in the coating. The increase in Co element content, along with the subsequent reduction in crystallite size of the deposited coating resulted in broader lattice peaks, thereby indicating a refined microstructure.

**Table 5:** Crystallite size by Scherrer’s equation of the coating with the optimized processing parameters.

Samples	hkl plane	2θ	θ	FWHM	Average crystallite size (D) / nm
Ni–Co–P	111	44.383	22.191	0.392	
	200	51.932	25.966	1.095	19.925
	220	76.567	38.283	0.417	
Ni–Co–P–BN(h)	111	44.474	22.237	0.517	
	200	51.751	25.875	1.204	16.687
	220	76.341	38.170	0.510	
Ni–Co–P–Al_2_O_3_	111	44.517	22.258	0.528	
	200	51.616	25.808	0.922	16.440
	220	76.325	38.162	0.518	

#### Microhardness of the coating

**Fig. 9:**
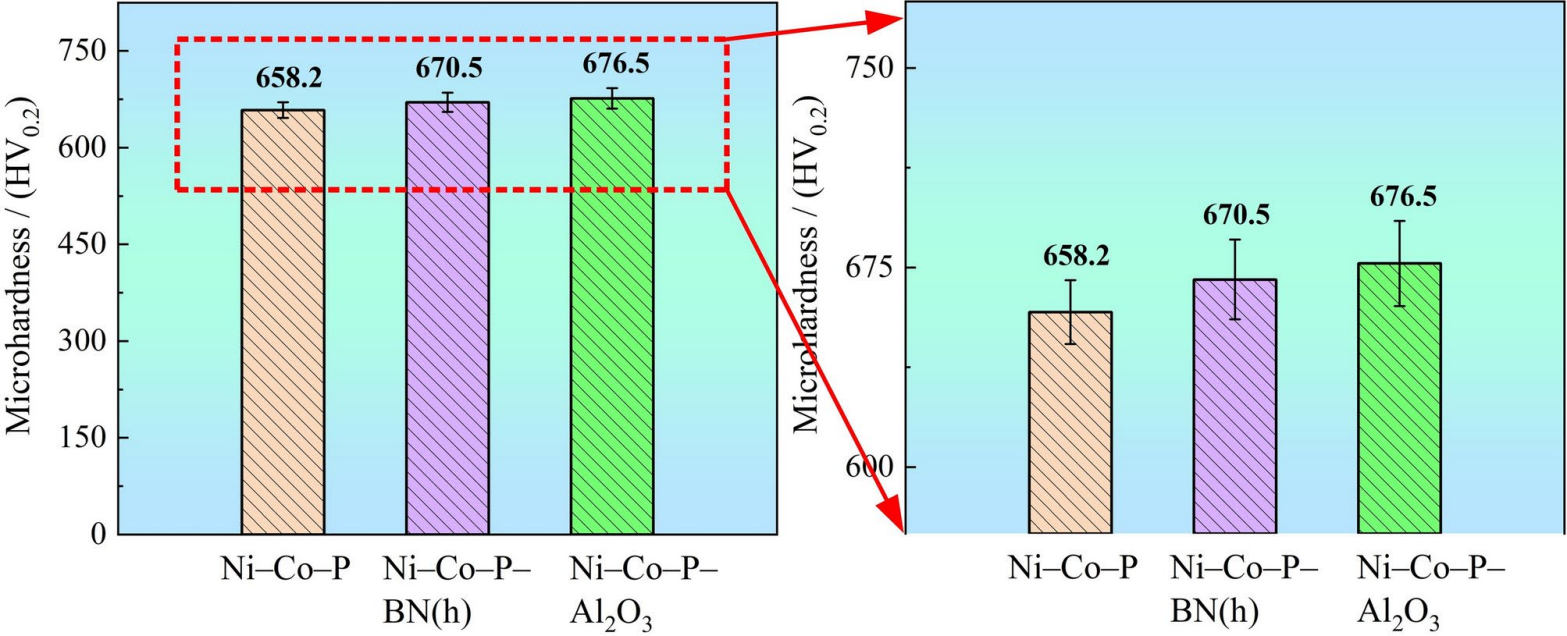
Microhardness of the Ni–Co–P, Ni–Co–P–BN(h) and Ni–Co–P–Al_2_O_3_ coating.

The microhardness of the Ni–Co–P, Ni–Co–P–BN(h), and Ni–Co–P–Al_2_O_3_ composite coatings produced under optimized processing parameters are illustrated in Fig. 9. The microhardness of the Ni–Co–P alloy coating was measured at 658.2 HV_0.2_, while the Ni–Co–P–BN(h) and Ni–Co–P–Al_2_O_3_ composite coatings exhibited microhardness values of 670.5 HV_0.2_ and 676.5 HV_0.2_, respectively. This demonstrated that the incorporation of BN(h) and Al_2_O_3_ nanoparticles into the coatings significantly enhanced their microhardness, such that all the nanocomposite coatings exhibit superior microhardness compared to the pure Ni–Co–P alloy coating. The observed increase in microhardness with addition of the BN(h) and Al_2_O_3_ nanoparticles was attributed to the synergistic effects of dispersive strengthening effect (uniform distribution) and grain refinement facilitated by the uniform distribution of BN(h) and Al_2_O_3_ nanoparticles within the metallic matrix. This hindered plastic deformation during loading, thereby increasing the load bearing capacity of the coatings, and constrain the growth of Ni–Co–P grains. Consequently, the nanocomposite coatings exhibit superior mechanical performance compared to pure Ni–Co–P alloy coatings.

**Fig. 10:**
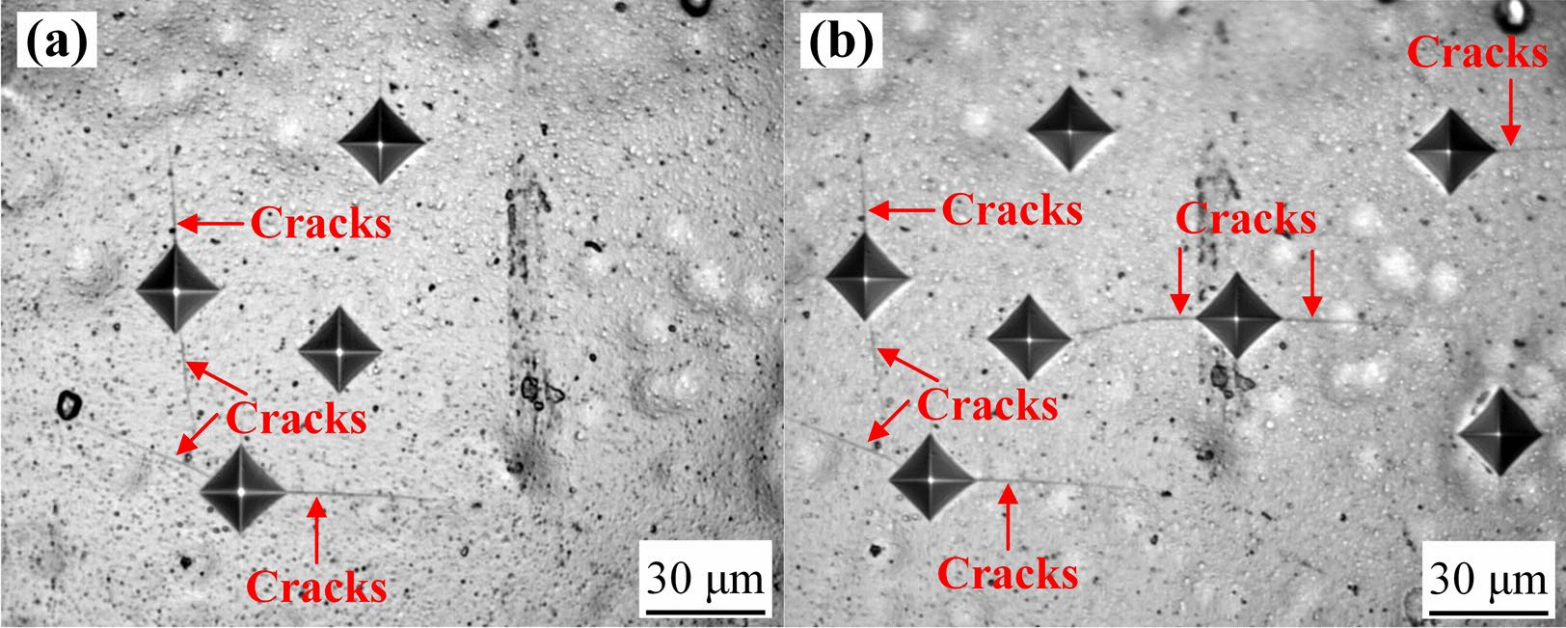
Optical microscopy image of the Vickers indent for the Ni–Co–P–Al_2_O_3_ coating.

It is reported that nanocomposite coating with lower grain sizes coupled with flatter and denser surface exhibited higher microhardness, which is the result of the combined effect of fine grain strengthening (Hall–Petch relationship) and dispersion strengthening (Orowan mechanism). Combined with Fig. 9 and Table 5, compared with the Ni–Co–P coating and Ni–Co–P–BN(h) nanocomposite coating, the Ni–Co–P–Al_2_O_3_ composite coating exhibited a smaller crystallite size. Consistent with previous findings, a reduction in the grain size on the sample surface results in an increased grain boundary, a smaller path for dislocation movements, greater resistance, and enhanced difficulty in slip transfer from one grain to another^56,57^. It is evident that the addition of BN(h) and Al_2_O_3_ nanoparticles further improve the microhardness of the coating. The optical microscopy image of the Vickers indent for the Ni–Co–P–Al_2_O_3_ nanocomposite coating is illustrated in Fig. 10. It can be seen that its shape was rhombus after the indenting. Additionally, the appearance of microcracks indicated that the deposited Ni–Co–P–Al_2_O_3_ composite coating were a kind of fracture toughness material. Therefore, it can be suggested that a load bearing Ni–Co–P–Al_2_O_3_ matrix exhibited limited effectiveness in resisting the formation and propagation of microcracks under the applied load.

#### wear resistance of the coating

**Fig.11:**
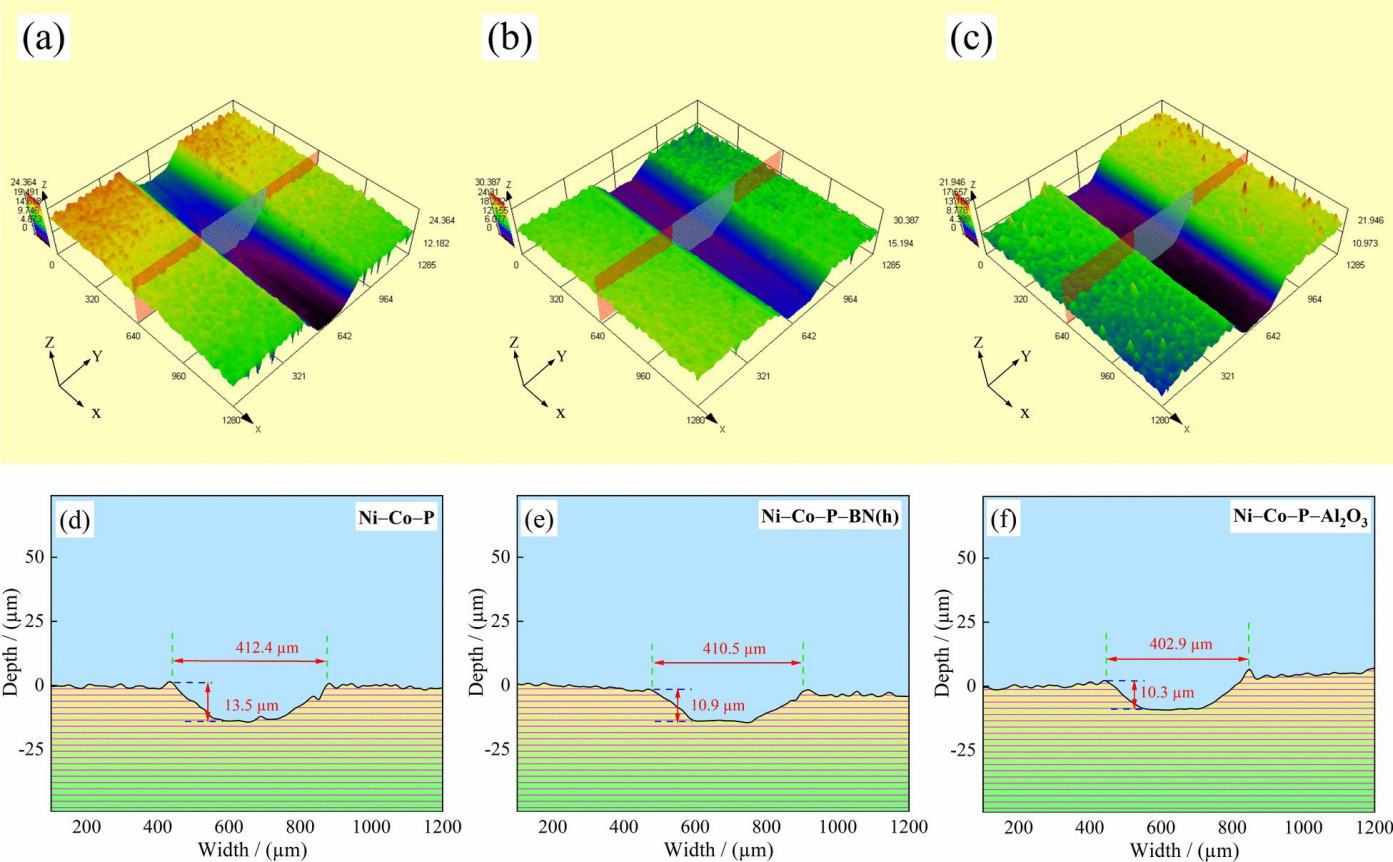
Laser scanning confocal microscopy (LSCM) three-dimensional (3D) images and two-dimensional (2D) cross-sectional profiles of worn surfaces of the coatings with the optimized processing parameters:

Wear resistance refers to the ability of a material to resist removal, surface damage, and deformation caused by mechanical actions^58^. Previous studies^59–61^ have established that wear resistance is affected by several factors such as the microstructure, microhardness, and friction coefficient of the coating. Notably, higher microhardness generally correlates with improved wear resistance^62^. Figure 11 exhibited the surface morphology, three-dimensional (3D) images, and two-dimensional (2D) cross-sectional profiles of worn surfaces of the coating. As shown in Fig. 11 (a) and (d), the wear scar width and depth of Ni–Co–P alloy coating was measured at 412.4 µm and 13.5 µm, respectively. Additionally, the scratch area of Ni–Co–P alloy coating appeared relatively flat, with the edges of the wear mark exhibiting a "hill-like" structure. Observations indicated coating peeling and accumulation phenomena. The primary wear mechanism for the Ni-Co-P alloy coating was identified as slight adhesive wear and abrasive wear. This was evident from the presence of plowing grooves and wear debris within the worn area. However, no microcracks, severe cracking, or large-scale peeling were observed, highlighting the coating’s good toughness and resistance to catastrophic failure under the given conditions.

As shown in Fig. 11 (b) and (e), the wear scar width and depth of Ni–Co–P–BN(h) nanocomposite coating were measured at 410.5 µm and 10.9 µm, respectively. These results indicate that the incorporation of BN(h) nano particles significantly enhanced wear resistance of composite coatings, even under optimized processing parameters. Additionally, the Ni–Co–P–BN(h) composite coating exhibited narrower and shallower wear scars compared to the Ni–Co–P alloy coating. Figure 11 (c) and (f) show the wear scar width and depth of the Ni–Co–P–Al_2_O_3_ nanocomposite coating, which were 402.9 µm and 10.3 µm, respectively.

(a, d) Ni–Co–P; (b, e) Ni–Co–P–BN(h); (c, f) Ni–Co–P–Al_2_O_3_.

The wear rate of the coatings was calculated on the basis of Eq. (4) ^63,64^. Where V is the wear volume (μm^3^), F is the load (N), L is the total wear distance (mm), S is the sectional area of the wear trace (μm^2^), D is the sliding length (μm), v is the reciprocating speed (mm/s), and T is the friction time (s).

4 $$Wear \, rate = \frac{V}{F \times L} = \frac{S \times D}{{F \times v \times T}}$$

**Fig.12:**
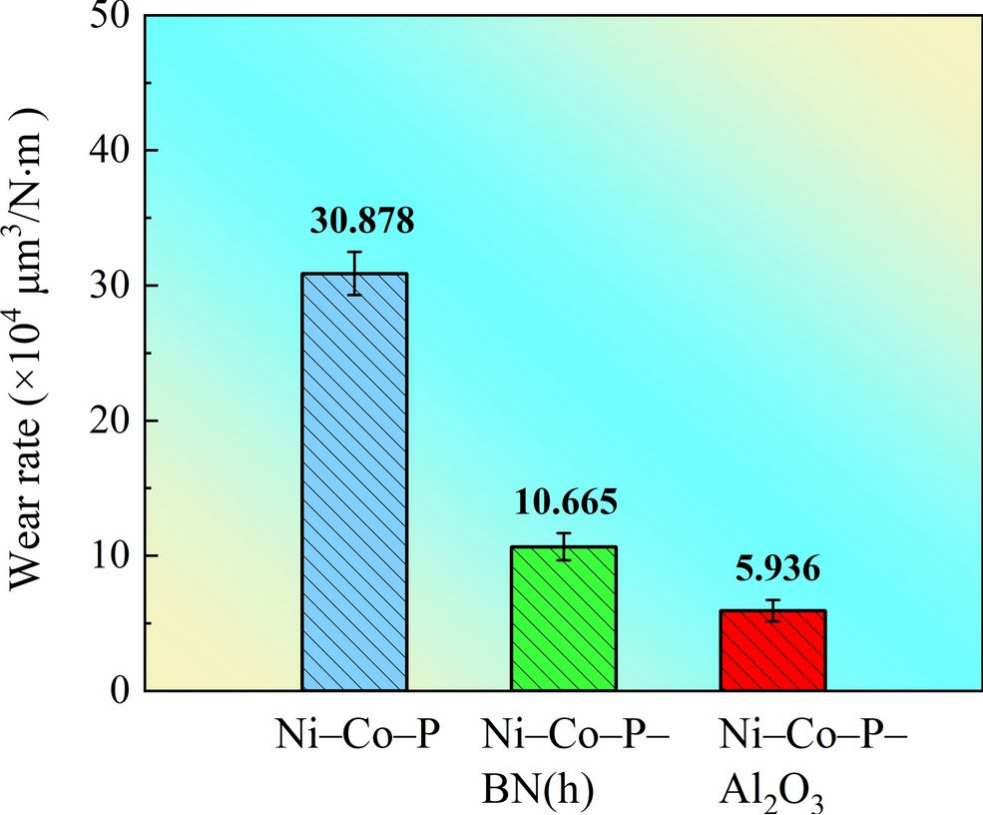
Wear rate of the coatings with the optimized processing parameters.

As shown in Fig. 12, the wear rates of the Ni–Co–P alloy coating and Ni–Co–P–BN(h) nanocomposite coating are 30.878 × 10^4^ and 10.665 × 10^4^ μm^3^/N·m, respectively. The wear rate results (Fig. 12) show that the Ni–Co–P–Al_2_O_3_ composite coatings prepared with the optimized processing parameters has a low wear rate (5.936 × 10^4^ μm^3^/N·m). This indicated that the Ni–Co–P–Al_2_O_3_ nanocomposite coating exhibited superior wear resistance (a smaller width, depth and wear rate) compared to Ni–Co–P alloy and Ni–Co–P–BN(h) nanocomposite coating. As such, it can be postulated that the inclusion of Al_2_O_3_ particles had a more pronounced effect on enhancing the wear resistance of the Ni–Co–P alloy coating than BN(h) nanoparticles.

Combined with the findings illustrated in Fig. 8 and Fig. 9, it is evident that the Ni–Co–P–Al_2_O_3_ composite coating demonstrated the highest microhardness coupled with smallest crystallite size, attributes which contributed to its superior wear resistance. In contrast, the wear of Ni–Co–P–BN(h) composite coatings was relatively more severe. of the worn surface of Ni–Co–P–BN(h) composite coatings displayed significant peeling and stacking phenomena due to combined effects of applied load and contact stress. Furrows, adhesive marks, and debris were observed in the worn area, which indicated that the dominant wear mechanism for Ni–Co–P–BN(h) composite coatings were slight adhesive wear and abrasive wear. On the other hand, the Ni–Co–P–Al_2_O_3_ composite coatings acted as an effective interface barrier during the wear process, effectively suppressing the deep propagation of cracks. This prevented the formation of micro cracks and cracking phenomena in the worn area, and no large-scale peeling occurred, indicating that the primary wear mechanism of Ni–Co–P–Al_2_O_3_ composite coating was abrasive wear.

It has been reported that nano particles can play a supporting and load-bearing role during the friction process, reducing the adhesive area on the friction surface. Notably, Al_2_O_3_ particles resist plastic deformation, hinder abrasive movement, and terminate the extension of wear marks during the friction process, thereby improving the ability of Ni–Co–P–Al_2_O_3_ composite coatings to resist adhesive and abrasive wear while delaying surface failure. Mameri et al.^65^ reported that nano Al_2_O_3_ enhanced the wear resistance of nickel matrix composites. Additionally, existing literature^66^ highlights that Al_2_O_3_ particles exerted the “nano-ball” effect in the friction process, which converted sliding friction into “rolling friction”. Similarly, Duan et al.^67^ reported that flaking nano Al_2_O_3_ in the friction process acted as abrasive grains and formed a lubricating film on the surface, which reduced direct metal-to-metal contact. Therefore, the Ni–Co–P–Al_2_O_3_ nanocomposite coatings prepared via jet electrodeposition exhibited excellent wear resistance under an optimal duty cycle.

**Fig.13:**
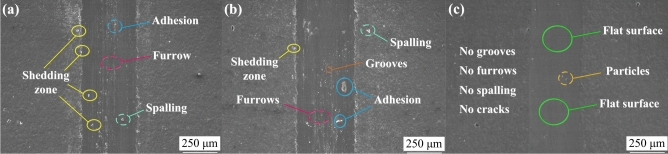
The worn morphology of the coatings prepared with the optimized processing parameters: (**a**) Ni–Co–P; (**b**) Ni–Co–P–BN(h); (**c**) Ni–Co–P–Al_2_O_3_.

Figure 13 depicts the micromorphology of the wear scars of Ni–Co–P, Ni–Co–P–BN(h), and Ni–Co–P–Al_2_O_3_ composite coatings, as observed through SEM under optimized processing parameters. In Fig. 13 (a), the Ni–Co–P coating exhibited prominent furrows and a significant shedding zone. Additionally, adhesion and spalling ere evident in the worn area, suggesting that the primary wear mechanism for the Ni–Co–P coating included brittle spalling, furrow formation, and abrasive wear. As shown in Fig. 13 (b), the wear on the Ni–Co–P–BN(h) composite coatings appeared relatively severe. Extensive peeling and stacking phenomena were observed on the friction surface, attributed to combined effects of load and contact stress. Furrows, adhesion, and debris were prevalent in the worn area, which indicated the wear mechanism for Ni–Co–P–BN(h) composite coatings was predominantly slight adhesive wear and abrasive wear. Figure 13 (c) highlights the wear characteristics of the Ni–Co–P–Al_2_O_3_ composite coatings. These coatings can act as an effective interface barrier during the wear process, significantly suppressing the deep propagation of cracks. Therefore, there were no micro cracks and cracking phenomena in the worn area, and no large-scale peeling occurred, indicating the wear mechanism of Ni–Co–P–Al_2_O_3_ composite coatings was mainly abrasive wear. The differences in wear mechanism between the Ni–Co–P–BN(h) and Ni–Co–P–Al_2_O_3_ composite coatings were the main reasons for the improved wear resistance.

**Fig. 14:**
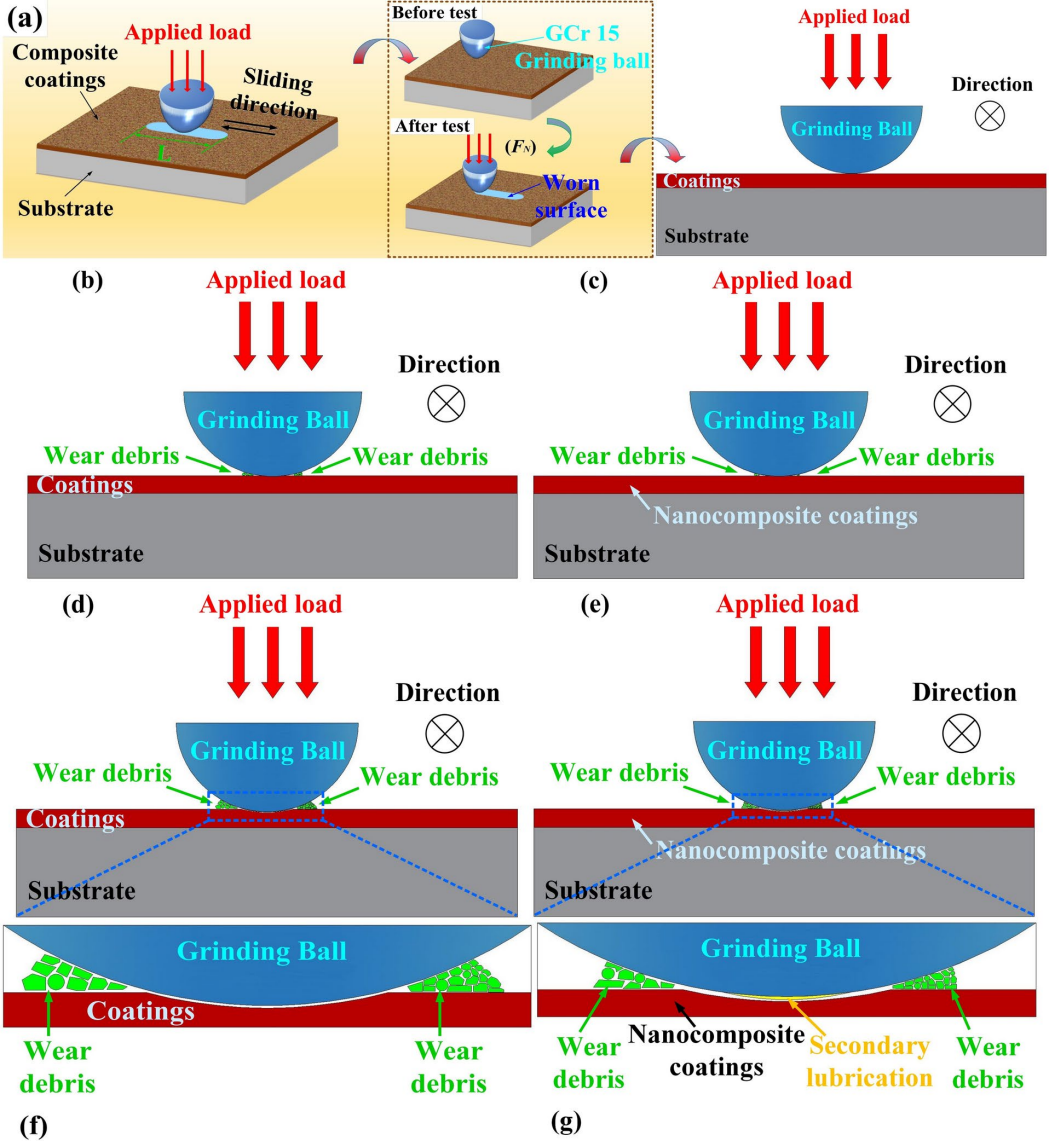
Schematic diagram of the wear mechanism of the Ni–Co–P alloy coating and nanocomposite coating.

To further analyze and discuss the tribological behavior of the Ni–Co–P coating and nanocomposite variants, a schematic diagram is presented in Fig. 14. Figure 14 (a) shows the experimental setup, where a reciprocating wear GCr15 grinding ball, subjected to a vertical force of 320 g, made direct contact with the coatings surface at room temperature (25 ± 1 ℃). The sliding motion of the grinding ball followed a linear reciprocating path. As shown in Fig. 14 (b) and (c), with increased wear time, wear debris accumulated on the surface of both the Ni–Co–P alloy coating and nanocomposite coatings. Notably, the nanocomposite coatings generate less wear debris initially, resulting in reduced friction during the early stages of wear. Subsequently, as wear progressed, as shown in Fig. 14 (d-g), friction films were produced in the abrasion area when the GCr15 grinding ball reciprocated. These friction films acted as solid lubricants on the wear surface, providing a secondary lubrication effect (see Fig. 14 (g)). This secondary lubrication mechanism was a significant factor contributing to the superior wear and frictional performance of the nanocomposite coatings.

## Conclusions

In this study, a novel Ni–Co–P alloy coating and Ni–Co–P nanocomposite coating were successfully prepared using jet electrodeposition. The influence of jet electrodeposition processing parameters (jet voltage, plating solution temperature, reciprocating sweep speed, jet gap, pulse frequency, and duty cycle) on the microhardness of the Ni–Co–P alloy coating was investigated. Mathematical models were developed, and the processing parameters were optimized by RSM. Furthermore, the cross-section morphology, EDS spectra, XRD patterns, microhardness and wear resistance of the coatings under optimal jet electrodeposition parameters was evaluated, and the main findings are summarized as follows:

(1) The BBD analysis results revealed that the established mathematical model was reliable. Based on the microhardness target response was a maximum value. The optimum Ni–Co–P alloy coating parameters optimized through the response surface method were as follows: jet voltage: 12.14 V, plating solution temperature: 61.60 °C, reciprocating sweep speed: 173.19 mm·s^−1^, jet gap: 2.05 mm, pulse frequency: 4.06 kHz and duty cycle: 0.81. At these parameters, the microhardness of the Ni–Co–P alloy coating was 656.7 HV_0.2_.

(2) Under the optimum jet electrodeposition parameters, SEM, EDS and XRD results revealed the significant influence of nano BN(h) and Al_2_O_3_ particles on the coatings’ thickness, Co element contents in the coating and crystallite size of Ni–Co–P nanocomposite coating. In addition, compared with the Ni–Co–P alloy coating and Ni–Co–P–BN(h) nanocomposite coating, Ni–Co–P–Al_2_O_3_ composite coating exhibited a larger thickness (18.16 µm), a higher Co element contents (39.51 wt·%) and a smaller crystallite size (16.440 nm).

(3) Under the optimum jet electrodeposition parameters, the microhardness and wear test results indicated that the addition of BN(h) and Al_2_O_3_ nanoparticles in the coatings can further improve the microhardness and wear resistance of Ni–Co–P alloy coating. Additionally, the microhardness of Ni–Co–P–BN(h) and Ni–Co–P–Al_2_O_3_ composite coatings were 670.5 HV_0.2_ and 676.5 HV_0.2_, respectively. The wear scar width of Ni–Co–P–BN(h) and Ni–Co–P–Al_2_O_3_ composite coatings were 410.5 µm and 402.9 µm, respectively. Therefore, Ni–Co–P–Al_2_O_3_ nanocomposite coatings exhibited excellent wear resistance.

## Data Availability

The datasets used and/or analysed during the current study available from the corresponding author on reasonable request.
